# Study on the influencing factors of bilateral high-frequency hearing loss in mining workers: The relationship between different occupational environmental pollutants and bilateral high-frequency hearing loss

**DOI:** 10.1371/journal.pone.0341728

**Published:** 2026-03-12

**Authors:** Zequn Shen, Zhe Yin, Aiyixianmuguli Tuerdi, Fen Wang, Danxia Sun, Xin Liu, Jing Zhang, Daoxian Ding, Xiaoxu Zhang

**Affiliations:** 1 School of Rehabilitation Nursing, Hangzhou Polytechnic University, Hangzhou, Zhejiang, China; 2 School of Public Health, Xinjiang Medical University, Urumqi, Xinjiang, China; 3 School of Humanities, Xinjiang Medical University, Urumqi, Xinjiang, China; 4 Xinjiang Uygur Autonomous Region Center for Disease Control and Prevention, Urumqi, Xinjing, China; 5 Hami Central Hospital, Hami, Xinjing, China; LSU Health Shreveport, UNITED STATES OF AMERICA

## Abstract

This retrospective case-control study analyzed the influencing factors of bilateral high-frequency hearing loss induced by the occupational environment in mining workers, and explored the effects of different occupational environmental pollutants on bilateral high-frequency hearing loss. Researchers collected basic information and occupational history of the research subjects through medical record retrieval. The research subjects were normal high-frequency hearing group and bilateral high-frequency hearing loss group, and different occupational environmental pollutants were grouped. Statistical analysis of influencing factors on bilateral high-frequency hearing loss and the impact of different occupational environmental pollutants on bilateral high-frequency hearing loss. The detection rate of bilateral high-frequency hearing loss among 2900 noise exposed workers was 14.17%; logistic regression multivariate analysis showed that gender, age, body mass index(BMI), and different occupational environmental pollutants were all influencing factors of bilateral high-frequency hearing loss(*P* < 0.05). The subgroup analysis results showed that different occupational environmental pollutants were still influencing factors for bilateral high-frequency hearing loss of mining workers in different groups such as males, age > 41 years, and normal BMI(*P* < 0.05). Men, increasing age, overweight and obesity were all risk factors for bilateral high-frequency hearing loss in workers exposed to occupational noise. Different occupational environmental pollutants had a more severe impact on bilateral high-frequency hearing loss, and different noise protection measures should be developed according to different occupational environmental pollutants.

## Introduction

With the continuous improvement of mechanization in mining operations, noise pollution has become an important occupational health issue in heavy industry operating environments. As a typical occupational group exposed to high noise, mining workers have particularly severe bilateral high-frequency hearing loss situations [1]. Hearing loss can result from a variety of factors, including noise, trauma, infection, aging, and so on [2]. Among these factors, noise exposure stands out as the most prevalent issue confronted by mining workers. Noise induced hearing loss is hearing damage caused by sudden exposure to pulse noise or long-term exposure to high-intensity noise [3]. The incidence rate of hearing loss caused by occupational noise exposure among Chinese workers was 36.6% [4]. In contrast, Indian workers had an occupational noise exposure hearing loss incidence rate of 50% [5]. Meanwhile, the prevalence of occupational noise exposure induced hearing loss in the United States is around 10% [6]. Bilateral high-frequency hearing loss not only weakened workers’ ability to identify environmental warning sounds and increased production safety risks, but also led to complications such as tinnitus and insomnia, seriously affecting the physical and mental health and quality of life of workers [7].

More research has focused on the correlation between mining noise and hearing loss, but there remains a lack of systematic exploration into how different occupational environmental pollutants, such as noise in combination with other factors, contribute to bilateral high-frequency hearing loss. In mining operation scenarios, noise and other pollutants often coexist in the production process. It was previously believed that hearing loss caused by the combined effect of noise and other pollutants was only attributed to noise exposure. However, in fact, noise is not the only factor causing occupational hearing loss; occupational exposure to various harmful substances could also damage hearing [8]. This article was based on the physical examination data of mining workers in Hotan, Xinjiang. First, explore the influencing factors of binaural high-frequency hearing loss in mining workers. Then, analyze the quantitative relationship between different occupational environmental pollutants and binaural high-frequency hearing loss. In summary, all of the above has practical significance for accurately formulating hearing protection strategies and optimizing mine noise management systems.

## 1 Materials and methods

### 1.1 Materials

**Fig 1:**
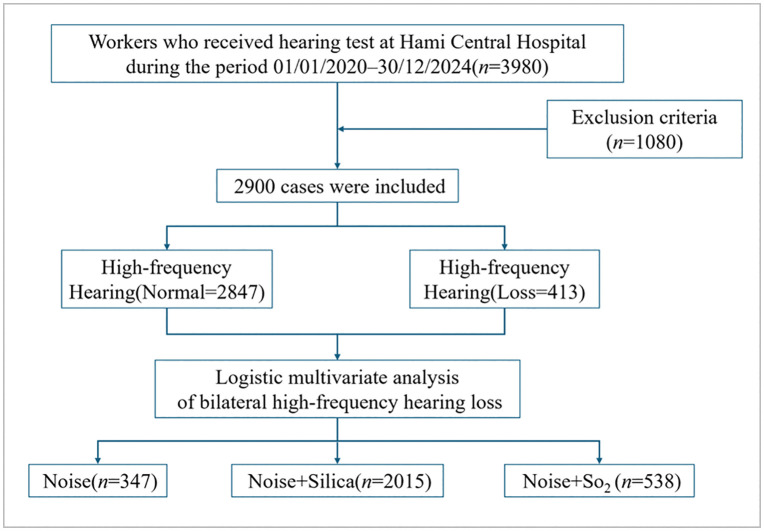
Study design flow chart.

A case-control study design was adopted in this study. The research subjects were workers exposed to occupational pollution who received physical examinations at Hami Central Hospital, Hami, Xinjiang, during the period 01/01/2020–30/12/2024. Data was exported between 01/01/2025 and 30/04/2025. Authors had access to information that could not identify individual participants during or after data collection. Clinical information about mining workers was extracted from the hospital information system (HIS). Information from HIS was compiled in abstracted evaluations. After screening according to inclusion and exclusion criteria, a total of 2900 cases were included, as shown in Fig 1. Inclusion criteria: (1) Conducted a physical examination at Hami Central Hospital; (2) Being currently employed; (3) Exposed to pollutants Noise, Noise+Silica(Sio_2_) or Noise+ Sulfur Dioxide(SO_2_) respectively. Exclusion criteria: (1) Incomplete medical examination data; (2) Noise exposure duration of less than 1 year; (3) A history of long-term ototoxic drug use, genetic hearing loss, traumatic hearing loss, or other confounding conditions affecting auditory function; (4) Diagnosis of severe immune system disorders. The working hours of physical examination workers were 8 hours per day, with 1–1.5 hours of rest between every 4 hours. Three groups took turns working within 24 hours, with two days off per week, but not all on weekends. This study was reviewed and approved by the Medical Ethical Review Committee of NIOPH, China CDC [NIOHP202421]

### 1.2 Methods

According to the requirements of China's GBZ188–2019 “Technical Specifications for Occupational Health Surveillance” and “GBZ492014 Diagnosis of Occupational Noise induced Hearing Loss”, pure tone hearing threshold tests were conducted on noise workers. Physicians conducted routine external ear examinations on the workers, including the auricle, external auditory canal, and eardrum. To eliminate the influence of temporary threshold shift (TTS), workers should be away from noisy work environment for 48 hours before physical examination. The otologists conducted audiological examination in a soundproof room with a noise background value<25 dB (A). During the audiometry process, background correction was performed, and pure tone air conduction hearing thresholds were measured at six frequencies of 500, 1000, 2000, 3000, 4000, and 6000 Hz in both ears for all workers. Age and gender corrections were also applied to the air conduction hearing thresholds of the workers. Calculation of high-frequency average hearing threshold for both ears:

BHFTA=(HLL+HLR)/6

BHFTA is the average high-frequency hearing threshold of both ears, measured in decibels(dB); HL_L_ and HL_R_ are the sum of the hearing levels of the left ear at 3000, 4000, and 6000 Hz, and the sum of the hearing levels of the right ear at 3000, 4000, and 6000 Hz, respectively, measured in decibels (dB). After diagnosis by professional doctors, it was determined that the average high-frequency hearing threshold of both ears was ≥ 40 dB, indicating bilateral high-frequency hearing loss.

### 1.3 Statistics

Exported occupational health examination results by medical record room staff, organized the data using Excel software, and analyzed the data using SPSS 25.0. Qualitative data were represented by cases (percentage) as n (%), the comparison between two groups used a chi-square test for a 2 × 2 contingency table, and the comparison between multiple groups used a chi-square test for an R × C contingency table; Using logistic regression to analyze the influencing factors of bilateral high-frequency hearing loss; The impact of different occupational environmental pollutants on bilateral high-frequency hearing loss was analyzed by subgroup analysis. A difference of *P* < 0.05 was considered statistically significant.

## 2 Results

### 2.1 Comparison of the basic conditions in bilateral high-frequency hearing loss and different occupational environmental pollutants

The majority of the research subjects were male (2244, 94.3%), with an average age of 37.79 ± 9.67 years. There were statistically significant differences (*P* < 0.05) in gender, age, body mass index(BMI), hypertension and left ventricular ejection fraction(LVEF) among different groups with bilateral high-frequency hearing loss and different occupational environmental pollutants, as shown in Table 1.

**Table 1:** Comparison of the basic conditions in bilateral high-frequency hearing loss and different occupational environmental pollutants (*n* = 2900).

Variables	High-frequency Hearing		*χ* ^*2*^	*P*	Pollutants			*χ* ^*2*^	*P*
	Normal	Loss			Noise	Noise+Silica	Noise+SO_2_		
**Gender**									
Male	2328(85.1)	409(14.9)	19.648	**<0.001**	316(11.5)	1928(70.4)	493(18.0)	21.264	**<0.001**
Female	159(97.5)	4(2.5)			31(19.0)	87(53.4)	45(27.6)		
**Age(years)**									
18 ~ 30	658(95.9)	28(4.1)	228.900	**<0.001**	60(8.7)	499(72.7)	127(18.5)	11.619	**0.020**
31 ~ 40	944(92.5)	76(18.4)			143(14.0)	696(68.2)	181(17.7)		
≥41	885(74.1)	309(25.9)			144(12.1)	820(68.7)	230(19.3)		
**BMI(kg/m**^**2**^)									
18.5 ~ 24.9	1169(88.0)	159(12.0)	16.986	**0.001**	120(9.0)	961(72.4)	247(18.6)	23.662	**<0.001**
<18.5	57(95.0)	3(5.0)			5(8.3)	49(81.7)	6(10.0)		
25.0 ~ 29.9	940(83.0)	192(17.0)			162(14.3)	767(67.8)	203(17.9)		
>30.0	180(84.5)	33(15.5)			32(15.0)	149(70.0)	32(15.0)		
**Smoke**									
No	809(84.3)	151(15.7)	1.957	0.162	125(13.0)	579(70.7)	156(16.3)	2.030	0.362
Yes	969(86.4)	152(13.6)			159(14.2)	803(71.6)	159(14.2)		
**Drink**									
No	897(85.7)	150(14.3)	0.069	0.792	128(12.2)	758(72.4)	161(15.4)	0.344	0.842
Yes	763(85.3)	132(14.7)			103(11.5)	658(73.5)	134(15.0)		
**Heart rate**									
Normal	127(85.2)	22(14.8)	0.289	0.791	24(16.1)	78(52.3)	47(31.5)	2.553	0.279
Abnormal	12(80.0)	3(20.0)			4(36.7)	9(60.0)	2(13.3)		
**Hypertension**									
No	2105(86.7)	323(13.3)	10.753	**0.001**	291(12.0)	1663(68.5)	474(19.5)	9.684	**0.008**
Yes	382(80.9)	90(19.1)			56(11.9)	352(74.6)	64(13.6)		
**Diabetes**									
No	78(84.8)	14(15.2)		0.999^*^	1(1.1)	79(85.9)	12(13.0)	/	/
Yes	10(90.9)	1(9.1)			/	11(100.0)	/		
**LVEF**									
Normal	1303(85.2)	226(14.8)	7.386	**0.007**	/	1039(68.0)	490(32.0)	11.751	**0.001**
Abnormal	116(76.8)	35(23.2)			/	123(81.5)	28(18.5)		

Missing values in BMI, Smoke, Drink, Heart rate, Hypertension, Diabetes, LVEF; *: Fisher’s exact probability method.

### 2.2 Comparison of noise exposure status in bilateral high-frequency hearing loss and different occupational environmental pollutants and

Statistically significant differences were observed in bilateral high-frequency hearing loss among workers with varying noise exposure years, noise intensity, and protection level(*P* < 0.05).The differences between different occupational environmental pollutants in enterprise scale, noise exposure years, noise intensity, and protection are statistically significant(*P* < 0.05), as shown in Table 2.

**Table 2:** Comparison of noise exposure status in bilateral high-frequency hearing loss and different occupational environmental pollutants(*n* = 2900).

Variables	High-frequency Hearing		*χ* ^*2*^	*P*	Pollutants			*χ* ^*2*^	*P*
	Normal	loss			Noise	Noise+Silica	Noise+So_2_		
**Enterprise scale**									
large	440(85.8)	73(14.2)	5.492	0.064	290(56.5)	139(27.1)	84(16.4)	1775.335	**0.001**
medium	1504(86.8)	229(13.2)			42(2.4)	1562(90.1)	129(7.4)		
small	543(83.0)	111(17.0)			15(2.3)	314(48.0)	325(49.7)		
**Noise exposure years**									
≤3	1401(88.3)	186(11.7)	78.672	**<0.001**	95(60.0)	1180(74.4)	312(19.7)	178.493	**<0.001**
4 ~ 15	849(86.8)	129(13.2)			185(18.9)	590(60.3)	203(20.8)		
16 ~ 30	190(73.6)	68(36.4)			64(24.8)	174(67.4)	20(7.8)		
>30	47(61.0)	30(39.0)			3(3.9)	71(92.2)	3(3.9)		
**Noise intensity(dB)**									
Electric(30 ~ 60)	559(91.5)	52(8.5)	40.291	**0.001**	25(4.1)	548(89.7)	38(6.2)	392.654	**0.001**
Construction(55 ~ 70)	218(88.6)	28(11.4)			/	121(49.2)	125(50.8)		
Mining(85)	1478(82.6)	312(17.4)			244(13.6)	1218(68.0)	328(18.3)		
Transportation(60 ~ 70)	232(91.7)	21(8.3)			78(30.8)	128(50.6)	47(18.6)		
**Protection**									
Yes	1478(82.6)	312(17.4)	38.930	**<0.001**	244(13.6)	1218(68.0)	328(18.3)	12.367	**0.002**
No	1009(90.0)	101(9.1)			103(9.3)	797(71.8)	210(18.9)		

### 2.3 The relationship between different occupational environmental pollutants and bilateral high-frequency hearing loss

**Fig 2:**
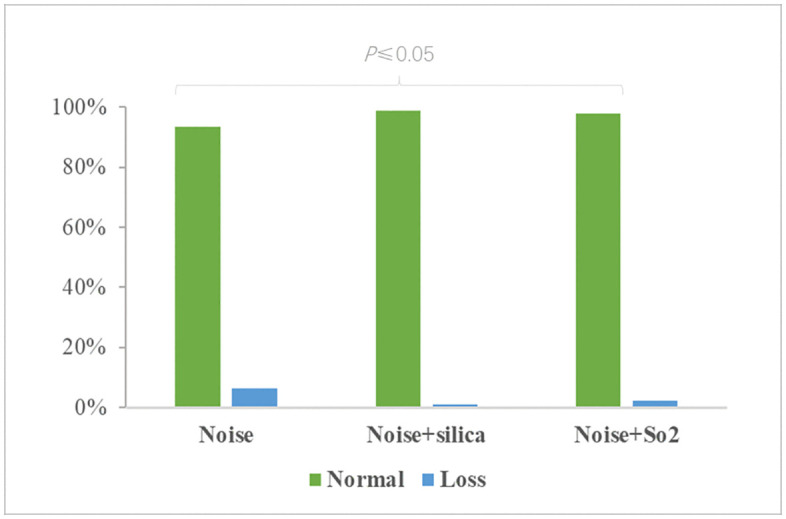
Analysis of the relationship between different occupational environmental pollutants and bilateral high-frequency hearing loss.

Different occupational environmental pollutants have an impact on bilateral high-frequency hearing loss (*P*< 0.05), as shown in Fig 2.

### 2.4 Logistic multivariate analysis of bilateral high-frequency hearing loss

In the multiple logistic regression model, the dependent variable was bilateral high-frequency hearing loss; The independent variables were those with *P* < 0.05 in univariate analysis, with “0” as the reference value. Due to multicollinearity between variables “Age” and " Noise exposure years “, " Noise intensity” and " Protection “, only “Age” and " Noise intensity " variables were retained in the model. For ordered level variables, dummy variables were set. The independent variable selection process was a stepwise regression method, and the regression coefficient test included *P* < 0.05. When excluded, the value was *P* < 0.10.

The model ultimately retained the independent variables: Gender, Age, BMI, and Pollutants. When the worker was female(*OR=0.135,95%CI:0.042 ~ 0.438*), the likelihood of bilateral high-frequency hearing loss was only 13.5% compared with males under the same conditions; The older the age, the greater the likelihood of bilateral high-frequency hearing loss among workers. Under the same conditions, workers between the ages of 30 and 40(*OR=1.740,95%CI:1.080* ~ *2.803*), as well as those over 40(*OR=7.342,95%CI:4.740* ~ *11.373*), were 1.74 times and 7.32 times more likely than workers under 30 to suffer from bilateral high-frequency hearing loss, respectively. Compared with workers who had normal BMI, overweight (*OR=1.138,95%CI:1.044* ~ 1.*689*) and obese workers(*OR=1.627,95%CI:1.054* ~ *2.512*) were more prone to bilateral high-frequency hearing loss, with the likelihood being 1.14 times and 1.62 times higher, respectively. Different types of noise pollutants had varying effects on bilateral high-frequency hearing loss in workers. Compared to working environments with individual noise pollution, workers in Noise+SO_2_ (*OR=2.117,95%CI:1.363* ~ *3.287*) working environment were more susceptible to bilateral high-frequency hearing loss, the likelihood of which was 2.12 times higher than that of workers with individual noise pollution, as shown in Table 3.

**Table 3:** Logistic multivariate analysis of bilateral high-frequency hearing loss.

Variables	*β*	*SE*	Wald*χ*^*2*^	*P*	*OR (95%CI)*
**Gender**					
female	−2.001	0.599	11.151	0.001	0.135 (0.042 ~ 0.438)
**Age(years)**					
31 ~ 40	0.554	0.243	5.182	0.023	1.740 (1.080 ~ 2.803)
≥41	1.994	0.223	79.711	<0.001	7.342 (4.740 ~ 11.373)
**BMI(kg/m**^**2**^)					
25.0 ~ 29.9	0.284	0.123	5.341	0.021	1.138 (1.044 ~ 1.689)
>30.0	0.487	0.222	4.825	0.028	1.627 (1.054 ~ 2.512)
**Pollutants**					
Noise+SO_2_	0.750	0.225	11.150	0.001	2.117 (1.363 ~ 3.287)

### 2.5 The relationship between different occupational environmental pollutants and bilateral high-frequency hearing loss under different influencing factors

Perform subgroup analysis based on gender, age, and BMI to further explore the impact of different occupational environmental pollutants on bilateral high-frequency hearing loss. The results showed that when the workplace pollutant was Noise + SO₂, males had a probability of bilateral high – frequency hearing loss that was 2.227 times (*OR*=2.227,*95%CI*:1.447 ~ 3.426) greater than when the workplace pollutant was Noise alone. Among workers aged 40, when the occupational environmental pollutant was Noise+SO₂, the likelihood of bilateral high-frequency hearing loss was 2.358 times (*OR*=2.358,*95%CI*:1.398 ~ 3.976) that of Noise alone. In normal weight workers with a BMI of 18.5 ~ 24.9, the probability of bilateral high-frequency hearing loss was 4.288 times(*OR*=4.288,*95%CI*:1.824 ~ 10.083) that when the occupational environmental pollutant was Noise+SO₂ compared with Noise alone, as shown in Table 4.

**Table 4:** The relationship between different occupational environmental pollutants and bilateral high-frequency hearing loss under different influencing factors.

variables	Pollutants	loss/ total	*P*	*OR(95%CI)*
**Gender**				
Male	Noise+SO_2_	106/493	**<0.001**	2.227 (1.447 ~ 3.426)
**Age(years)**				
≥41	Noise+SO_2_	77/230	**<0.001**	2.358 (1.398 ~ 3.976)
**BMI(kg/m**^**2**^)				
18.5 ~ 24.9	Noise+SO_2_	48/247	**<0.001**	4.288 (1.824 ~ 10.083)

## 3 Discussion

The mining operational environment is marked by a diverse array of pollutants, coupled with high levels of exposure intensity and prolonged durations of exposure. Workers in this setting are often subjected to long-term exposure to multiple pollutants, including noise, chemical toxins, and dust, rendering them highly vulnerable to irreversible bilateral high-frequency hearing loss. This study has revealed that the development of bilateral high-frequency hearing loss among mining workers is affected by several factors, namely gender, age, BMI, as well as various types of occupational environmental pollutants.

### 3.1 Male mining workers were more likely than female mining workers to suffer from bilateral high-frequency hearing loss

In this study, a notable gender disparity was observed in the incidence of bilateral high-frequency hearing loss, with statistical significance (*P*< 0.05). Specifically, the prevalence of this condition among female mining workers was merely 13.5% of that reported among their male counterparts. According to existing literature, such differences stem from variations in job nature, which could result in males being exposed to higher cumulative doses of noise compared to females. Additionally, distinct job responsibilities contribute to a gender-based imbalance in noise exposure levels during mining activities. Men were more frequently assigned to frontline roles, including underground mining, excavation, and mechanical operation, where they faced direct and prolonged exposure to high-intensity noise sources such as pneumatic picks, crushers, and ventilation equipment. The noise levels in these environments often surpassed 85dB, coupled with relatively extended periods of exposure [9]. In contrast, women were predominantly positioned in ground support roles, such as scheduling and quality inspection, where the intensity of noise exposure typically remained below 80dB, and their exposure times were more fragmented [10].

Estrogen exerts a protective effect on the female auditory system [11]. Specifically, estrogen can enhance microcirculation within the inner ear and boost blood flow perfusion to the cochlea. Concurrently, it plays a role in regulating ion channel function, thereby maintaining a stable cochlear potential [12,13]. Moreover, there exists a gender bias regarding awareness of hearing protection and the adoption of preventive measures. Studies have shown that male mining workers are less likely to wear earplugs or earmuffs than their female counterparts, a difference attributed to the high work intensity they experience [14]. Additionally, some men perceive that protective gear impedes communication or diminishes work efficiency, whereas women tend to be more conscientious about adhering to safety regulations, thereby minimizing their direct exposure to noise.

### 3.2 The older the mining workers, the greater the likelihood of bilateral high-frequency hearing loss

In this study, it was observed that the likelihood of bilateral high-frequency hearing loss increased with the age of mining workers. This phenomenon is attributed to the synergistic impact of multiple factors. As miners age, there is a natural decline in physiological functions, which render them more susceptible to hearing damage. Additionally, the cumulative effect of occupational exposure to noise and other harmful factors becomes more pronounced over time, further exacerbating the risk of hearing loss. Moreover, differences in health management practices across various age stages also contribute to hearing impairment among mining workers.

As individuals age, the human auditory system is prone to degenerative transformations [15]. Specifically, the hair cells within the cochlea may experience a reduction in quantity and a decline in functional integrity due to the natural aging process [16,17]. This degeneration is particularly pronounced in the basal region of the cochlear basement membrane, which is highly sensitive to high-frequency sounds. Consequently, elderly workers gradually exhibit a diminished capacity to perceive high-frequency sounds, and in severe cases, this auditory decline may even be associated with an increased risk of vascular dementia [18,19].

Older mining workers often have longer work experience and longer exposure to noise, which can lead to accumulated damage over time. Studies indicate that coal mine workers with over 15 years on the job face a hearing loss rate that is more than 3.5 times higher than that of their counterparts with 10–14 years of experience [20]. Notably, the risk of high-frequency hearing loss escalates to a high-risk category after 25 years of work, whereas the danger of noise-induced deafness reaches a critical level following 30 years of occupational noise exposure [21].

From the perspectives of health management and individual behavior, older miners may tend to ascribe their hearing issues to the natural aging process, leading them to overlook early symptoms and consequently miss the optimal window for intervention. In addition, some elderly workers have reduced the frequency of using hearing protection equipment due to inconvenient wearing and poor comfort, resulting in continuous exposure of their ears to hazardous noise levels [22]. The progression of age is frequently accompanied by chronic conditions such as hypertension and diabetes, which may indirectly exacerbate hearing loss by impairing inner ear microcirculation [23].

### 3.3 Overweight and obesity are risk factors for bilateral high-frequency hearing loss in mining workers

This study reveals that coal miners with a body mass index (BMI) of 24 kg/m² or higher exhibit significantly elevated high-frequency hearing thresholds compared to their normal-weight counterparts. This observed phenomenon is intricately linked to metabolic disturbances, disruptions in inner ear blood circulation, and the synergistic effects of occupational exposure.

Overweight and obesity often occur alongside metabolic abnormalities, including insulin resistance and dyslipidemia. Hyperinsulinemia can trigger a chronic inflammatory reaction in inner ear tissue by activating inflammatory factors TNF-α and IL-6, subsequently damaging cochlear hair cells and auditory nerves [24,25]. Concurrently, the dyslipidemia associated with overweight and obesity speeds up the process of atherosclerosis of the blood vessel walls in the inner ear, reducing the blood supply to the cochlea and causing hypoxia injury to hair cells, especially in the apical region of the cochlear basement membrane. A prospective cohort study spanning 7 years showed that the HR for hearing loss at 1 kHz were 1.21 (95%CI:1.08 ~ 1.36) and 1.66 (95%CI:1.33 ~ 2.08) for those with BMI 25.0–29.9 kg/m^2^ and BMI >= 30.0 kg/m^2^, respectively, compared to individuals with BMI < 25.0 kg/m^2^. For hearing loss at 4 kHz, the corresponding HR were 1.14 (95%CI:1.05, ~ 1.23) and 1.29 (95%CI:1.09 ~ 1.52) [26].

Furthermore, within mining settings, overweight and obesity have a synergistic effect with occupational exposure factors, thereby affecting hearing [27]. Due to their higher physical burden and limited mobility, obese workers often encounter greater difficulties in appropriately and consistently wearing hearing protection equipment, thereby exposing themselves to higher actual noise doses. Moreover, the physical decline associated with obesity also renders workers more prone to fatigue during strenuous operations, diminishing their capacity to tolerate noise hazards [28]. Additionally, obese workers tend to have increased sweat gland secretion, which can lead to discomfort and stuffiness when wearing protective earmuffs, prompting them to reduce the duration of use. This, in turn, further escalates the risk of hearing loss.

### 3.4 Mining workers working in Noise+SO_2_ environment have more severe bilateral high-frequency hearing loss compared to workers working in pure noise environment

The differences in the effects of different occupational environmental pollutants on bilateral high-frequency hearing loss in this study were statistically significant(*P*< 0.05); After subgroup analysis of relevant influencing factors, different pollutants still have an impact on bilateral high-frequency hearing loss in mining workers, and the difference is statistically significant(*P*< 0.05). Exposure to composite pollutants is more likely to accelerate the process of hearing loss compared to simple noise exposure, and its intrinsic correlation needs to be analyzed from multiple dimensions. The above differences are attributed to the synergistic effects of chemical toxicity, exposure dose-response relationships, the superposition of pathophysiological mechanisms, and the specificity of exposure pathways, which need to be analyzed from multiple perspectives for their inherent correlations.

Noise and other pollutants exert synergistic toxic effects. Noise damages the inner ear blood labyrinth barrier, making cochlear hair cells more susceptible to chemical or particulate pollutants [29]. After entering the human body, silica tends to deposit in the lungs through the respiratory tract, triggering an inflammatory response that can release a vast array of cytokines. These cytokines can be circulated through the bloodstream and affect the inner ear, thereby exacerbating noise induced oxidative stress and cell apoptosis [30]. As a strong irritant gas, SO₂ not only directly damages the respiratory epithelium but also dissolves in bodily fluids to form sulfite and sulfuric acid. These substances can enter the inner ear through blood circulation and thus disrupt the acid-base balance of lymphatic fluid, interfere with the ion channel function of hair cells, and accelerating the degeneration of high-frequency capillary cells together with noise [31]. The differences in the pathways of pollutants affect the damage patterns. From the perspective of chemical toxicity, SO₂ and noise have a stronger synergistic damage effect on hearing. In contrast, silica dust mainly indirectly affects hearing through physical deposition and inflammatory reactions, and the speed and intensity of its damage are not as high as those caused by the chemical toxicity of SO₂.

The dose-response relationship of exposure further highlights the hazards of composite pollution. Research has shown that workers who are exposed to both noise and SiO₂ have a higher elevation of hearing threshold when compared with workers exposed only to noise [32]. When noise is combined with SO₂ exposure, it can also enhance the damage that noise inflicts on cochlear nerve fibers, and the hearing loss is more significant. Furthermore, at the pathological level, the combination of noise and SO₂ triggers more severe oxidative stress and inflammatory reactions. Exposure to SO₂ can induce the production of large amounts of reactive oxygen species (ROS) in the body, activate inflammatory pathways such as NF-κB, and promote the release of inflammatory factors such as TNF-α and IL-6 [33]; Exposure to noise can also lead to an increase in oxidative stress levels in the inner ear tissue, and the combination of the two further exacerbates cochlear hair cell damage, accelerating the irreversible decline of high-frequency hearing. The particularity of the exposed path also amplifies the harm of Noise+SO₂ [34]. Both noise and SO₂ can directly affect the auditory system. In contrast, the exposure of silica dust mainly occurs through respiratory deposition, which indirectly affects the inner ear after triggering systemic reactions through pulmonary inflammation, and its damage has a certain degree of delay. In addition, the water solubility of SO₂ makes it easier to adhere to the surface of protective equipment, reducing the effectiveness of hearing protection products such as earplugs and earmuffs, thereby further increasing the actual exposure dose of workers.

## 4 Conclusion

For older mining workers, trade unions and medical examination hospitals should provide appropriate health education to attract sufficient attention from older workers for early examination and prevention. Older mining workers with chronic diseases should actively manage chronic diseases and take medication according to medical advice to avoid cumulative damage to their high-frequency hearing. We should implement gender-specific management for high noise positions based on gender differences in mining workers with bilateral high-frequency hearing loss, reducing long-term high-intensity exposure for men in these positions. Furthermore, we should conduct specialized training on hearing protection for men, enforce the standardized use of protective equipment, and enhance compliance with noise protection measures in the workplace. Overweight and obese workers should implement a dynamic job rotation mechanism based on work intensity and noise exposure levels when arranging their work, appropriately shorten the continuous working time in environments with high-frequency noise, and ensure that their hearing has sufficient recovery time. In addition, equip overweight and obese workers with hearing protection products that better fit their head and ear characteristics. Customize enlarged earplugs or adjustable ear cushions to ensure that the protective equipment can effectively attenuate high-frequency noise. Install efficient gas collection and purification devices to reduce the concentration of So₂ in the air and regularly monitor the SO₂ concentration in the work environment to ensure compliance with national occupational exposure limits. At the same time, design the ventilation system in a reasonable manner to ensure effective circulation of fresh air and reduce the duration of combined exposure to SO₂ and noise. Strengthen health promotion and training education to enhance workers’ self-protection awareness and ability.

## 5 Limitations

Although current research has revealed the association between age, gender, BMI, and different workplace pollutants with high-frequency hearing loss in coal miners, there are still limitations. This is a retrospective case-control study, and causality cannot be directly inferred from it. Meanwhile, as the data of the study subjects were sourced from hospital physical examination reports, although bias control has been carried out in the setting of inclusion and exclusion criteria and the application of statistical methods, uncontrollable selection bias still exists. In the future, a long-term tracking occupational health monitoring system can be established. Through large sample cohort analysis and longitudinal tracking, we will explore the cumulative effects of age, gender, obesity, and noise induced hearing loss, as well as the causal effects among these factors and with bilateral high-frequency hearing loss. At the same time, the analysis of the composite effects of pollutants, as well as the dynamic monitoring of the composite pollution effects involving pollutants SiO₂ and SO₂ under long – term exposure to noise, should be conducted.

## References

[1] Chadambuka A, Mususa F, Muteti S. Prevalence of noise induced hearing loss among employees at a mining industry in Zimbabwe. Afr Health Sci. 2013;13(4):899–906. doi: 10.4314/ahs.v13i4.6 24940310 PMC4056470

[2] Michels TC, Duffy MT, Rogers DJ. Hearing Loss in Adults: Differential Diagnosis and Treatment. Am Fam Physician. 2019;100(2):98–108. 31305044

[3] Rikhotso O, Morodi TJ, Masekameni DM. Occupational Health and Safety Statistics as an Indicator of Worker Physical Health in South African Industry. Int J Environ Res Public Health. 2022;19(3):1690. doi: 10.3390/ijerph19031690 35162712 PMC8835012

[4] Yang P, Xie H, Li Y, Jin K. The Effect of Noise Exposure on High-Frequency Hearing Loss among Chinese Workers: A Meta-Analysis. Healthcare (Basel). 2023;11(8):1079. doi: 10.3390/healthcare11081079 37107914 PMC10137611

[5] Basu S, Aggarwal A, Dushyant K, Garg S. Occupational Noise Induced Hearing Loss in India: A Systematic Review and Meta-Analysis. Indian J Community Med. 2022;47(2):166–71. doi: 10.4103/ijcm.ijcm_1267_21 36034244 PMC9400345

[6] Dobie RA. The burdens of age-related and occupational noise-induced hearing loss in the United States. Ear Hear. 2008;29(4):565–77. doi: 10.1097/AUD.0b013e31817349ec 18469718

[7] Jayakody DMP, McIlhiney P, Stegeman I, Eikelboom RH. A cross-sectional study of how high-frequency hearing loss impacts cognitive functions in middle-aged-to-older adults. Front Aging Neurosci. 2025;17:1560307. doi: 10.3389/fnagi.2025.1560307 40357231 PMC12066433

[8] Kuang D, Yu YY, Yang Y, Gao Y, Tu C, Wang L. High frequency hearing loss detection rate in occupational noise exposed workers in China: a Meta-analysis. Zhonghua Lao Dong Wei Sheng Zhi Ye Bing Za Zhi. 2021;39(3):184–9. doi: 10.3760/cma.j.cn121094-20200306-00106 33781033

[9] Wang X, Kang N, Dong Y, Liu K, Ning K, Bian H, et al. Noise exposure assessment of non-coal mining workers in four provinces of China. Front Public Health. 2023;10:1055618. doi: 10.3389/fpubh.2022.1055618 36699889 PMC9870050

[10] Sam WY, Anita AR, Hayati KS. Prevalence of hearing loss and hearing impairment among small and medium enterprise workers in Selangor, Malaysia. JSM. 2017;46(02):267–74. doi: 10.17576/jsm-2017-4602-11

[11] Thwaites SJ, van den Buuse M, Gogos A. Differential effects of estrogen and testosterone on auditory sensory gating in rats. Psychopharmacology (Berl). 2014;231(1):243–56. doi: 10.1007/s00213-013-3231-5 23929132

[12] Williamson TT, Zhu X, Pineros J, Ding B, Frisina RD. Understanding hormone and hormone therapies’ impact on the auditory system. J Neurosci Res. 2020;98(9):1721–30. doi: 10.1002/jnr.24588 32026519

[13] Charitidi K, Meltser I, Tahera Y, Canlon B. Functional responses of estrogen receptors in the male and female auditory system. Hear Res. 2009;252(1–2):71–8. doi: 10.1016/j.heares.2008.12.009 19450435

[14] Andresen NS, Coreas S, Villavisanis DF, Lauer AM. Comparison of age-related pigmentary changes in the auditory and vestibular systems within mouse and human temporal bones. Front Neurosci. 2021;15:680994. doi: 10.3389/fnins.2021.680994 34054423 PMC8163230

[15] Fuchs PA, Lauer AM. Efferent inhibition of the Cochlea. Cold Spring Harb Perspect Med. 2019;9(5):a033530. doi: 10.1101/cshperspect.a033530 30082454 PMC6496333

[16] Kolla L, Kelly MC, Mann ZF, Anaya-Rocha A, Ellis K, Lemons A, et al. Characterization of the development of the mouse cochlear epithelium at the single cell level. Nat Commun. 2020;11(1):2389. doi: 10.1038/s41467-020-16113-y 32404924 PMC7221106

[17] Vgontzas AN, Bixler EO, Chrousos GP. Obesity-related sleepiness and fatigue: the role of the stress system and cytokines. Ann N Y Acad Sci. 2006;1083:329–44. doi: 10.1196/annals.1367.023 17148748

[18] Ringen K, Dement JM, Quinn P, Cloeren M, Chen A, Cranford K, et al. Hearing impairment and tinnitus among older construction workers employed at DOE facilities. Am J Ind Med. 2022;65(8):644–51. doi: 10.1002/ajim.23406 35726605

[19] Shore SE, Roberts LE, Langguth B. Maladaptive plasticity in tinnitus--triggers, mechanisms and treatment. Nat Rev Neurol. 2016;12(3):150–60. doi: 10.1038/nrneurol.2016.12 26868680 PMC4895692

[20] Griffiths TD, Lad M, Kumar S, Holmes E, McMurray B, Maguire EA, et al. How Can Hearing Loss Cause Dementia?. Neuron. 2020;108(3):401–12. doi: 10.1016/j.neuron.2020.08.003 32871106 PMC7664986

[21] Erol İ. Investigation of Occupational Noise-Induced Hearing Loss of Underground Coal Mines. Mining, Metallurgy & Exploration. 2022;39(3):1045–60. doi: 10.1007/s42461-022-00585-1

[22] Ruan Y, Huang G, Zhang J, Mai S, Gu C, Rong X, et al. Risk analysis of noise-induced hearing loss of workers in the automobile manufacturing industries based on back-propagation neural network model: a cross-sectional study in Han Chinese population. BMJ Open. 2024;14(5):e079955. doi: 10.1136/bmjopen-2023-079955 38760055 PMC11103207

[23] Naicker K. Noise-induced hearing loss and hearing protection: Attitudes at a South African coal mine. S Afr J Commun Disord. 2024;71(1):e1–12. doi: 10.4102/sajcd.v71i1.966 38299534 PMC10839152

[24] Chadha S, Kamenov K, Cieza A. The world report on hearing, 2021. Bull World Health Organ. 2021;99(4):242-242A. doi: 10.2471/BLT.21.285643 33953438 PMC8085630

[25] Shi X, Dong Y, Li Y, Zhao Z, Li H, Qiu S, et al. Inflammasome activation in mouse inner ear in response to MCMV induced hearing loss. J Otol. 2015;10(4):143–9. doi: 10.1016/j.joto.2015.12.001 29937798 PMC6002578

[26] Zhang Y, Jin W, Zhang D, Lin C, He H, Xie F, et al. TNF-α Antagonizes the Effect of Leptin on Insulin Secretion through FOXO1-Dependent Transcriptional Suppression of LepRb in INS-1 Cells. Oxid Med Cell Longev. 2022;2022:9142798. doi: 10.1155/2022/9142798 35198097 PMC8860543

[27] Hu H, Tomita K, Kuwahara K, Yamamoto M, Uehara A, Kochi T, et al. Obesity and risk of hearing loss: A prospective cohort study. Clin Nutr. 2020;39(3):870–5. doi: 10.1016/j.clnu.2019.03.020 30954364

[28] Amaro J, Ubalde-López M, Lucas R. History of work-related health problems in a population-based sample of women: An exploratory factor analysis. Work. 2021;68(3):563–76. doi: 10.3233/WOR-203394 33612504

[29] Canis M, Bertlich M. Cochlear capillary pericytes. In: Birbrair A, editor. 2019. p. 115–23. 10.1007/978-3-030-11093-2_7 30937866

[30] Hu A, Li R, Chen G, Chen S. Impact of respiratory dust on health: a comparison based on the toxicity of PM2.5, Silica, and Nanosilica. Int J Mol Sci. 2024;25(14):7654. doi: 10.3390/ijms25147654 39062897 PMC11277548

[31] Park M, Han J, Jang M-J, Suh M-W, Lee JH, Oh SH, et al. Air pollution influences the incidence of otitis media in children: A national population-based study. PLoS One. 2018;13(6):e0199296. doi: 10.1371/journal.pone.0199296 29953484 PMC6023207

[32] Myshchenko I, Pawlaczyk-Luszczynska M, Dudarewicz A, Bortkiewicz A. Health risks due to co-exposure to noise and respirable crystalline silica among workers in the open-pit mining industry-results of a preliminary study. Toxics. 2024;12(11):781. doi: 10.3390/toxics12110781 39590961 PMC11598117

[33] Zhang L, Yi H, Sang N. Sulfur dioxide-induced exacerbation of airway inflammation via reactive oxygen species production and the toll-like receptor 4/nuclear factor-κB pathway in asthmatic mice. Toxicol Ind Health. 2021;37(9):564–72. doi: 10.1177/07482337211033136 34448417

[34] Zhang Y, Liu Y, Li Z, Liu X, Chen Q, Qin J, et al. Effects of coexposure to noise and mixture of toluene, ethylbenzene, xylene, and styrene (TEXS) on hearing loss in petrochemical workers of southern China. Environ Sci Pollut Res Int. 2023;30(11):31620–30. doi: 10.1007/s11356-022-24414-6 36449247

